# Distribution of HLA-A, -B and -DRB1 Genes and Haplotypes in the Tujia Population Living in the Wufeng Region of Hubei Province, China

**DOI:** 10.1371/journal.pone.0038774

**Published:** 2012-06-14

**Authors:** Li Zhang, Dangxiao Cheng, Ning Tao, Min Zhao, Fan Zhang, Yulin Yuan, Xiaoping Qiu

**Affiliations:** 1 Institute of Virology, Medical School of Wuhan University, Wuhan, Hubei, China; 2 University of Toronto, Toronto, Ontario, Canada; 3 Institute of Biophysics, Chinese Academy of Sciences, Beijing, China; 4 Zhongnan Hospital, Wuhan University, Wuhan, Hubei, China; 5 Department of Dissection, Medical School of Wuhan University, Wuhan, Hubei, China; Central China Normal University, China

## Abstract

**Background:**

The distribution of HLA alleles and haplotypes varies widely between different ethnic populations and geographic areas. Before any genetic marker can be used in a disease-associated study it is therefore essential to investigate allelic frequencies and establish a genetic database.

**Methodology/Principal Findings:**

This is the first report of HLA typing in the Tujia group using the Luminex HLA-SSO method HLA–A, –B and -DRB1 allelic distributions were determined in 124 unrelated healthy Tujia individuals, and haplotypic frequencies and linkage disequilibrium parameters were estimated using the maximum-likelihood method. In total 10 alleles were detected at the HLA–A locus, 21 alleles at the HLA–B locus and 14 alleles at the HLA-DRB1 locus. The most frequently observed alleles in the HLA-I group were HLA–A*02 (35.48%), A*11 (28.23%), A*24 (15.73%); HLA–B*40 (25.00%), B*46 (16.13%), and B*15 (15.73%). Among HLA-DRB1 alleles, high frequencies of HLA-DRB1*09 (25.81%) were observed, followed by HLA-DRB1*15 (12.9%), and DRB1*12 (10.89%). The two-locus haplotypes at the highest frequency were A*02–B*46A (8.47%), followed by A*11–B*40 (7.66%), A*02–B*40 (8.87%), A*11–B*15 (6.45%), A*02–B*15 (6.05%), B*40–DRB1*09 (9.27%) and B*46–DRB1*09 (6.45%). The most common three-locus haplotypes found in the Tujia population were A*02–B*46–DRB1*09 (4.84%) and A*02–B*40–DRB1*09 (4.03%). Fourteen two-loci haplotypes had significant linkage disequilibrium. Construction of a neighbor-joining phylogenetic tree and principal component analysis using the allelic frequencies at HLA-A was performed to compare the Tujia group and twelve other previously reported populations. The Tujia population in the Wufeng of Hubei Province had the closest genetic relationship with the central Han population, and then to the Shui, the Miao, the southern Han and the northern Han ethnic groups.

**Conclusions/Significance:**

These results will become a valuable source of data for tracing population migration, planning clinical organ transplantation, carrying out HLA-linked disease-associated studies and forensic identification.

## Introduction

The Tujia ethnic minority is one of the main minority groups in China. Its population ranks number six, just after the Zhuang, Manchu, Hui, Miao and Uygur among all the 56 Chinese ethnic minorities. The Tujia is an ancient ethnic group who have inhabited a narrow region bordering Hunan, Hubei, Sichuan and Guizhou provinces since the Qin dynasty [1]. The Tujia have played an important role in China’s economic and social development. The Tujia ethnic minority is normally recognized as comprising two subgroups which are the south branch Tujia and the north branch Tujia according to their geographic distribution and their minority culture origination. The south branch Tujia mainly inhabit areas in Chongqing and east of Guizhou province while the north branch Tujia mainly inhabit En-Shi, Hubei Province and XiZhou, Hunan province. The Wufeng Tujia autonomous county, located in the southwest of Hubei province, covers an area of 2372 square kilometers and their total population of 208,000 is 67% Tujia. The whole county is located in a branch of the Wuling Mountains and 86.3% of it is mountain area with an average elevation of 500 meters above sea level. Because of the characteristic isolated geographical distribution of Wufeng Tujias, they are able to live in this region and maintain their unique original ethnic culture, making this a very valuable resource for the study of familial genetics and research into inherited diseases [2].

The human leukocyte antigen (HLA) system, a group of closely linked genes occupying 1/3000^th^ of the human genome, resides on the short arm of human chromosome 6 (6p21.3) and spans about 3.5 to 4.0 kilobase pairs. Genes in the HLA complex are categorized into three basic groups: class I, class II and class III. HLA-A and HLA-B belong to the HLA class I heavy chain group, whereas HLA-DRB1 belongs to the HLA class II Beta chain group. The HLA gene family is the most complicated immunogenetic system which also has the highest rate of polymorphism among human genes [3]. These highly variable HLA polymorphisms present as allelic and haplotype differences between different ethnic groups, nationalities and even residents of different regions [4]. Research on HLA polymorphism has the following benefit. 1) By using higher frequency HLA antigens one can easily find HLA antigen-matched donors and recipients among people without a blood relationship. If genes of a high frequency HLA antigen are accidentally found within a haplotype, it will be much easier to find a corresponding donor or recipient. 2) Because products encoded by different alleles may present their antigen or react to antigen differently, this cause different allele carriers to exhibit different immune reactions to the same pathogen so that they will have a different immune reaction to some diseases. If some key alleles are present at a very high frequency in some ethnic groups or residents of some particular region, this might be the cause of a high incidence of some diseases in those ethnic groups or areas. 3) By analyzing variations in HLA allele frequency we will be able to understand the development of races and the origination of different ethnic groups.

**Figure 1:**
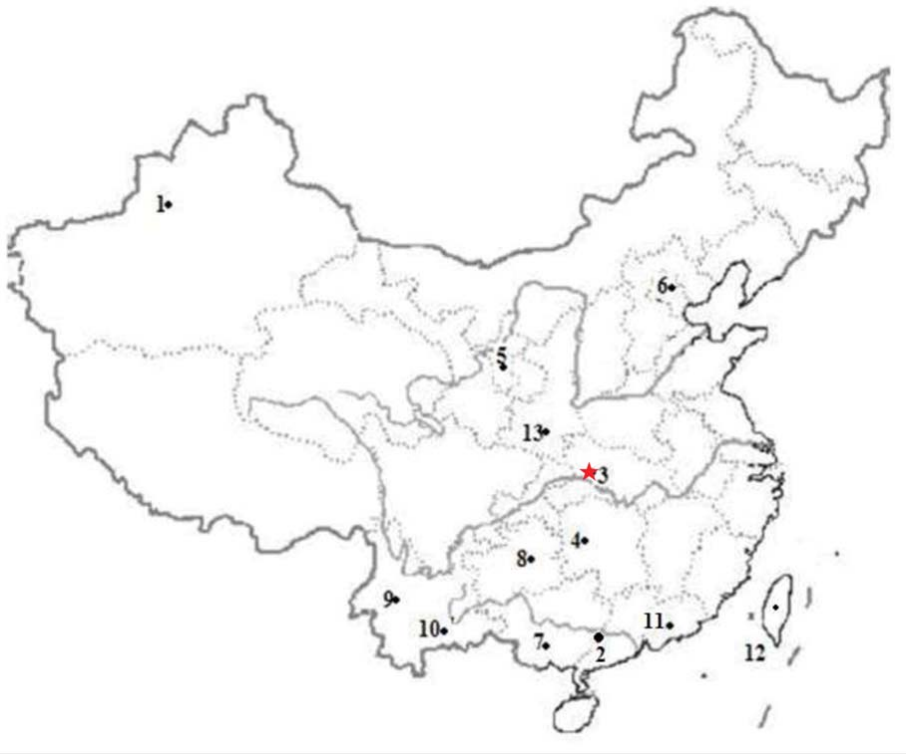
Map showing the sites of the populations used in this study. 1,Uyghur 2,Southern-Han 3,Tujia 4,Miao 5,Hui 6,Northern-Han 7,Bouyei 8,Shui 9,Bulang 10,Hani 11,Minnan 12,Taiwan-Aborigines 13,Middle-Han.

In this study, one hundred twenty-four non-blood relationship Tujia from Wufeng, Hubei province were recruited and their HLA alleles classified into HLA–A, –B or –DRB1 loci alleles using a WAKFlow HLA typing kit (Luminex HLA-SSO; Luminex HLA-SSO Inc., Shanghai, China) on the Multi-Analyte Profiling system (xMAP). The allele and haplotype frequency of HLA loci were calculated and their allele frequencies were compared with those of other ethnic groups (Fig. 1). The aims of this study were: 1) To outline the heritage status of HLA–A, –B and DRB1 loci in the Tujia inhabiting the Wufeng region. 2) To study the cause of genetic heterogeneity among Wufeng Tujia and the stage of the Wufeng Tujia among other ethnic groups in the whole process of human evolution. This study will help us to further understand the genetic background of HLA–A, –B and DRB1 loci alleles and their relationship to disease in the Wufeng Tujia population.

**Figure 2:**
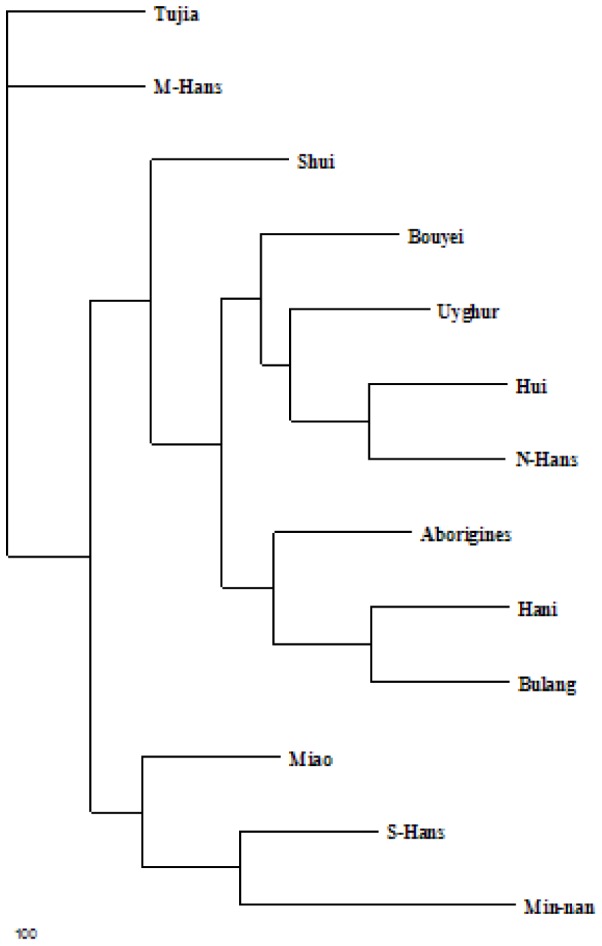
Phylogeny based on HLA allele frequencies. Dendroram constructed by the neighbor-joining method showing the relationship between Tujia populations in Hubei with other populations in Chinese individuals of 12 regions based on the frequencies of HLA-A loci.

**Figure 3:**
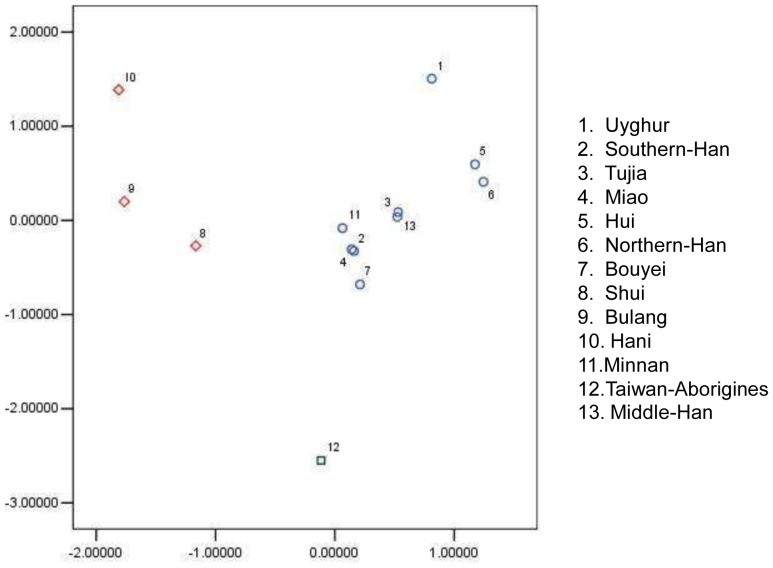
Result of Principal component analysis. Principal component analysis between Tujia population in Hubei with Chinese other 12 ethnic group based on the allelic frequencies of HLA-A loci.

## Results

### Hardy–Weinberg Tests of HLA-A, -B and -DRB1 Loci

The *p* values of the HLA–A, –B, and DRB1 loci in Hardy-Weinberg equilibrium tests were 0.88, 0.139839, and 0.711948, respectively (see Table 1). These results show that the HLA allelic distribution in the Wufeng region Tujia is in Hardy-Weinberg equilibrium at these loci.

**Table 1:** Observed and expected heterozygosity and Hardy–Weinbergsignificance for human leukocyte antigen-A, -B, and -DRB1loci in Tujia ethnicity.

Locus	Observed heterozygosity	Expected heterozygosity	P value
A	0.77419	0.76378	0.88
B	0.92742	0.87048	0.139839
DRB1	0.85484	0.87178	0.711948

### Genetic Polymorphisms of the HLA-A, -B, and -DRB1 Loci

The allelic frequency distributions of the HLA-A, -B and -DRB1 loci in the Tujia population of Wufeng region are shown in Table 2. In all, 10 alleles were identified at the HLA-A locus, 21 alleles at the HLA-B locus, and 14 alleles at the HLA-DRB1 locus in the 124 individuals of the Tujia population analyzed. In this population, four HLA–A alleles, A*02, A*11, A*24 and A*33, had frequencies greater than 5% with a cumulative frequency of 86.3%. Five HLA-B alleles, B*40, B*46, B*15, B*13 and B*51, had frequencies greater than 5%, with a cumulative frequency of 72.19%. Eight HLA-DRB1 alleles, DRB1*09, DRB1*15, DRB1*12, DRB1*04, DRB1*08, DRB1*11, DRB1*14 and DRB1*13, had frequencies greater than 5% and their cumulative frequency was 90.73%. HLA–A*02, A*11 and A*24 were the most common HLA–A alleles (frequencies were 35.48%, 28.23% and 15.73% respectively), whereas at the HLA–B locus the alleles with the most common frequency were B*40 (25%), B*46 (16.13%) and B*15 (15.73%) and at the HLA-DRB1 locus, the most common alleles were DRB1*09 (25.81%), DRB1*15 (12.9%) and DRB1*12 (10.89%).

**Table 2:** Allelic frequencies of HLA–A, B and DRB1 loci in Tujia population living in Wufeng region, China.

HLA–A	Allelic Frequency	HLA–B	Allelic Frequency	HLA–DRB1	Allelic Frequency
A*01	2.02%	B*07	2.02%	DRB1*01	2.02%
A*02	35.48%	B*13	9.68%	DRB1*04	9.27%
A*03	1.21%	B*15	15.73%	DRB1*07	3.63%
A*11	28.23%	B*27	0.40%	DRB1*08	8.87%
A*24	15.73%	B*35	1.21%	DRB1*09	25.81%
A*26	2.82%	B*37	1.61%	DRB1*3	0.4%
A*30	2.42%	B*38	1.21%	DRB1*10	1.61%
A*31	4.84%	B*39	3.63%	DRB1*11	8.47%
A*33	6.86%	B*40	25.00%	DRB1*12	10.89%
A*68	0.40%	B*44	2.02%	DRB1*13	6.05%
		B*46	16.13%	DRB1*14	8.47%
		B*48	1.21%	DRB1*15	12.9%
		B*50	0.81%	DRB1*16	1.21%
		B*51	5.65%	DRB1*17	0.4%
		B*52	2.42%		
		B*53	0.40%		
		B*54	3.63%		
		B*55	2.02%		
		B*56	0.40%		
		B*58	4.44%		
		B*67	0.40%		

### HLA Haplotype Frequency and Linkage Disequilibrium

We found 63 HLA–A–B haplotypes; 86 HLA-B-DRB1 haplotypes and 145 HLA-A-B-DRB1 haplotypes. There were 22 types of HLA–A–B haplotype, 31 types of HLA–B-DRB1 haplotype, and 20 types of HLA–A-B–DRB1 haplotype, with a frequency greater than 1%, and with cumulative frequencies of 77.84%, 72.56% and 36.28%, respectively, as shown in Table 3. In the Tujia population, the most common HLA-A-B haplotypes were A*02–B*46A (8.47%), A*11–B*40 (7.66%), A*02–B*40A (8.87%), A*11–B*15 (6.45%) and A*02–B*15 (6.05%) and the most common B-DRB1 haplotypes were B*40–DRB1*09 (9.27%) and B*46–DRB1*09 (6.45%). The most common HLA-A-B-DRB1 haplotypes were A*02–B*46–DRB1*09 (4.84%) and A*02–B*40–DRB1*09 (4.03%). To analyze the haplotype frequency and significant linkage disequilibrium parameters of HLA two-loci haplotypes in the Tujia population of Wufeng region, China, we calculated the linkage disequilibrium parameter of two loci haplotypes. In the two loci haplotypes, HLA-A*33-B*58, HLA-A*02–B*35, A*24–B*56, A*68–B*53, A*02–B*27, A*11–B*50, A*24–B*67, HLA-B*56–DRB1*14, B*37–DRB1*10, B*50–DRB1*07, B*58–DRB1*17, B*67–DRB1*15, B*27–DRB1*04 and HLA-B*53–DRB1*11 had significant linkage disequilibrium (relative linkage disequilibrium parameter 1). The significant linkage disequilibrium parameters of two-loci haplotypes are shown in Table 4.

**Table 3:** Main high-frequency HLA haplotypes and their frequencies of Tujia population (haplotype frequency ≥1%).

HLA A-B	HaplotypeFrequency	HLA B-DRB1	HaplotypeFrequency	HLA A-B-DRB1	HaplotypeFrequency
A*02–B*39	2.02%	B*37- DRB1*10	1.61%	A*11- B*39- DRB1*08	1.21%
A*24–B*40	4.84%	B*40- DRB1*08	2.42%	A*11- B*13- DRB1*09	1.61%
A*11–B*39	1.61%	B*39- DRB1*08	1.21%	A*11- B*46- DRB1*12	2.02%
A*02–B*46	8.47%	B*40- DRB1*09	9.27%	A*11- B*15- DRB1*15	2.42%
A*02–B*13	3.63%	B*46- DRB1*14	2.02%	A*02- B*46- DRB1*09	4.84%
A*02–B*40	8.87%	B*13- DRB1*04	1.21%	A*31- B*51- DRB1*15	1.21%
A*11–B*15	6.45%	B*13- DRB1*11	1.61%	A*24- B*40- DRB1*09	1.21%
A*11–B*40	7.66%	B*15- DRB1*12	2.42%	A*33- B*58- DRB1*13	1.61%
A*33–B*58	4.44%	B*40- DRB1*13	1.61%	A*02- B*40 -DRB1*09	4.03%
A*11–B*13	4.44%	B*13- DRB1*09	2.02%	A*02- B*15- DRB1*09	2.02%
A*11–B*46	4.03%	B*46- DRB1*12	4.03%	A*24- B*40- DRB1*15	1.61%
A*02–B*15	6.05%	B*15- DRB1*08	1.21%	A*02- B*15- DRB1*11	1.61%
A*02–B*35	1.21%	B*40- DRB1*12	1.61%	A*11- B*40- DRB1*15	1.21%
A*24–B*46	2.42%	B*13- DRB1*12	1.61%	A*02- B*40- DRB1*08	1.21%
A*31–B*40	1.21%	B*15- DRB1*15	2.42%	A*11- B*40- DRB1*09	1.61%
A*31–B*51	2.42%	B*46- DRB1*09	6.45%	A*02- B*40- DRB1*04	1.61%
A*24–B*51	1.21%	B*13- DRB1*07	1.61%	A*11- B*15- DRB1*04	1.61%
A*02–B*54	1.21%	B*39- DRB1*15	1.21%	A*24- B*46- DRB1*12	1.21%
A*24–B*15	2.02%	B*46- DRB1*08	1.21%	A*24- B*15- DRB1*09	1.21%
A*24–B*55	1.21%	B*15- DRB1*09	3.63%	A*26- B*40- DRB1*09	1.21%
A*30–B*13	1.21%	B*40- DRB1*14	2.02%		
A*26–B*40	1.21%	B*51- DRB1*15	1.61%		
		B*54- DRB1*14	1.61%		
		B*58- DRB1*13	1.61%		
		B*40- DRB1*11	1.21%		
		B*15- DRB1*04	2.82%		
		B*51- DRB1*09	1.61%		
		B*40 DRB1*15	4.03%		
		B*54- DRB1*09	1.21%		
		B*15- DRB1*11	2.42%		
		B*40- DRB1*04	2.02%		
Total	77.84%		72.56%		36.28%

**Table 4:** Haplotype frequency and significant linkage disequilibrium parameter of HLA two-loci haplotypes in Tujia population of Wufang region, China.

HLA A–B	HF	D′	HLA B-DRB1	HF	D′
A*33–B*58	0.044355	100%	B*56- DRB1*14	0.004032	100%
A*02–B*35	0.012097	100%	B*37- DRB1*10	0.016129	100%
A*24–B*56	0.004032	100%	B*50- DRB1*07	0.008065	100%
A*68–B*53	0.004032	100%	B*58- DRB1*17	0.004032	100%
A*02–B*27	0.004032	100%	B*67- DRB1*15	0.004032	100%
A*11–B*50	0.008065	100%	B*27 -DRB1*04	0.004032	100%
A*24–B*67	0.004032	100%	B*53- DRB1*11	0.004032	100%
A*03–B*07	0.008065	0.66	B*48- DRB1*15	0.008065	0.61728
A*24–B*48	0.008065	0.60	B*38- DRB1*08	0.008065	0.63422
A*24–B*55	0.012097	0.53	B*35- DRB1*04	0.008065	0.63259
			B*40- DRB1*16	0.008065	0.55556

Abbreviations: HF, haplotype frequency D′, relative linkage disequilibrium parameter.

### Construction of a Phylogenetic Tree

The phylogenetic tree shown in Figure 2 was constructed using the allelic frequencies at the HLA-A locus of the Tujia population in Wufeng and other ethnic groups. We compared the Tujia population (number of individuals analyzed:124) to the Uyghur (n = 104) [5], southern Han (n = 172), northern Han (n = 152), central Han (n = 211), Min-nan (n = 7137), Taiwan aborigines (n = 111), Miao (n = 154) [6], Hui (n = 122) [7], Shui (n = 153), Bouyei (n = 109) [8], Bulang (n = 116) and Hani (n = 150) [9].The phylogenetic tree is shown in Fig. 2. The genetic structure of the Tujia is closest to the central Han population.

### Principal Component Analysis

Principal component analysis of the 13 ethnic groups was based on the allelic frequencies of the HLA-A locus shown in Fig. 3.The results show that all the ethnic groups can be divided into three clusters. The first is the Bulang and Hani. The second is the Taiwan aborigines. The remaining ethnic groups were in the top right quadrant.

## Discussion

Reliable data on HLA allele frequency is a basic necessity for research into individual recognition and population genetics. We investigated the distribution of HLA alleles and haplotypes in the Wufeng Tujia population. A total of 10, 21 and 14 alleles were observed at the HLA-A, -B and HLA-DRB1 loci respectively. The high frequency alleles at the HLA-A and -B and DRB1 loci in the Tujia are shown in Table 2, and are similar to the high frequency alleles distributed among the Han population who inhabit the north, central and south of China. At HLA-A, -B and DRB1 loci, the presentation of high frequency alleles in the northern Han are higher than those of the southern Han; the presentation of those high frequency alleles exhibits some differences among different populations of Han who inhabit the south, central and north of China and some of those alleles, such as A*01, A*03, A*23, A*26, A*29, A*30, A*31, A*32, A*33, A*66 A*68, B*07, B*13, B*27, B*35, B*37, B*39, B*44, B*50, B*51 and B*52, show a trend towards a frequency decrease from north to south corresponding to the location of the population; however other alleles, such as B*38, B*48, B*53, B*54, B*55 and B*56, have a reverse trend of frequency distribution with a decrease from southern to northern populations. The results of our statistical analysis showed that allele frequency at HLA-A*03 (*p* = 0.0334), HLA-A*11 (*p* = 0.0043), HLA-A*30 (*p* = 0.0004), HLA-B*15 (*p* = 0.0388), HLA-B*35 (*p* = 0.0011), HLA-B*40 (*p* = 0.0001), HLA-B*46 (*p* = 0.0001), DRB1*07 (*p* = 7.33×10^5^), DRB1*08 (*p* = 0.0023), DRB1*09 (*p* = 0.0028)and DRB1*15 (*p* = 0.0219) is significantly different between the Tujia and the northern Han (*p*<0.05). Comparison of the Tujia with the southern Han revealed a significant allele frequency difference at loci HLA-A*31 (*p* = 0.01502), HLA-B*38 (*p* = 0.0191), HLA-B*40 (*p* = 0.0473), HLA-B*52 (*p* = 0.0180), DRB1*01 (*p* = 0.0162), DRB1*03 (*p* = 0.0077), DRB1*09 (*p* = 0.0480) and DRB1*13 (*p* = 0.0499) (*p*<0.05); however, significant allele frequency differences were only found at loci HLA-A*31(*p* = 0.0165), HLA-A*30 (*p* = 0.0070), DRB1*07 (*p* = 0.0305) and DRB1*09 (*p* = 0.0256) in comparison between the Tujia and the central Han (*p*<0.05). In comparison with the central Han, the Tujia have a similar allele frequency distribution at HLA-A, -B and DRB1 gene groups, while at HLA-B loci the allele frequency distribution is even with no significant difference between the two ethnic groups.

A significant linkage disequilibrium of the following haplotypes HLA-A*33–B*58, HLA-A*02–B*35, HLA-A*24–B*56, A*68–B*53, A*02–B*27, A*11–B*50, A*24–B*67, HLA-B*56–DRB1*14, HLA-B*37–DRB1*10, B*50–DRB1*07, B*58–DRB1*17, B*67–DRB1*15, B*27–DRB1*04 and B*53–DRB1*11 was identified in the Tujia population. This means that an HLA-matched donor from the non-blood-related donor pool can easily be found if a patient who carries these haplotypes needs treatment involving hematopoietic stem cell transplantation. This linkage disequilibrium among HLA genes is also very important for research involving data analysis on HLA-related diseases. In a comparison of the most common haplotypes of HLA-A-B-DRB1 in the Tujia with those of the northern, central and southern Han, the frequency distribution of A*02–B*46–DBR1*09 is higher than in the southern Han and much higher than the northern Han. All these results show that HLA loci and haplotypes in the Tujia have significant genetic polymorphisms.

Genetic distance is a method of comparing overall evolutionary divergence between two populations which normally relates to factors such as history, geology and language. Although they are under strong selective pressure, HLA genes have been successfully used in genetic distance research. The Tujia ethnic group is one of the most important minor ethnic nationalities in China. In comparison to other nationalities, the Tujia have unique ethnic characteristics, customs, culture and lifestyle due to the influence of geography and other factors. On the other hand, during the historic development of the Tujia nationality they have had a close relationship with different ethnic groups from outside areas; this has caused systemic genetic development of the Tujia due to their communication within their own groups as well as with outside ethnic groups. Therefore the study of population genetics in re-exploring and explaining the relationship of the Tujia nationality to other populations is very significant. In order to identify the origins of genetic heterogeneity in the Tujia who reside in Wufeng, Hubei province, we compared their allele frequency at HLA gene loci with twelve other ethnic groups. Genetic distances were computed, dendrograms were constructed using the neighbor-joining method and principal component analysis was carried out.

Genetic distance here refers to the genetic divergence between populations within a species. A small genetic distance indicates a close genetic relationship between two populations whereas a large genetic distance indicates a distant genetic relationship [10]. The genetic relationship between the Tujia and the other twelve ethnic groups studied was based on the allelic frequency at the HLA-A locus shown in Figure 2. In the Tujia, the result of genetic analysis at HLA-B and -DRB1 loci showed the same trend as that at the HLA-A locus, which is why we selected allelic frequency at the HLA-A locus as the basis for genetic distance analysis in this study Fig. 2 shows that the Tujia and the central Han are the most distinct groups while the remaining eleven groups can be divided into two clusters; Cluster one: Miao, Taiwan-minnan/southern Han; cluster two: the remaining eight ethnic groups. The northern Han share a cluster with the Hui. The Tujia population was most closely related to the central Han, and then to the Shui, the Miao, the southern Han and the northern Han. This study showed that the Tujia of Hubei province (the northern branch of the Tujia minority) have very similar haplotypes and allele frequency to the central Han; this indicates that the two population groups have a similar blood relationship and population genetics structure. Principal component analysis of Taiwan aborigines, Shui, Bulang and Hani populations were placed at the furthest distance from the Tujia. This might be due to the four ethnic populations living in relatively isolated areas and thus having limited genetic communication with other populations.

The Tujia ethnic nationality belongs to Tibeto-Burman group. According to studies on the origin of the Tujia ethnic nationality, Pan Guang Dan [11] was the first researcher who thought that the Tujia are the descendants of the ancient Ba people who inhabited the area bordering Huan, Hubei, Sichuang and Geizhong provinces. The Ba ethnic nationality, an ancient ethnic group who inhabited southwest China, formed and got its name around the time of the Xia (2100–1600 BC) and Shang (1600–1046 BC) dynasties and developed after the later Shang dynasty developed into the Northern and Southern Dynasties (420–589 AD). According to written records, as early as the later Shang dynasty, the Ba people had established their tribal territory, called Ba Guo. Around 611 BC, the Chu allied with the Ba Guo and Qing and extinguished Yong Guo. Subsequently Ba Guo became one of the strongest countries and regularly fought with Chu Guo for several hundred years. Around 316 BC, the Qing conquered Ba Guo. During the later era Qing Dynasty (1636–1911 AD), the government started a new policy of replacing local tribal leaders with government officers to administrate the local Ba people. This policy enabled huge numbers of Han and Miao people to enter and reside in the Tujia populated areas thus expanding the gene pool for the Tujia [12]. In fact, communications between the Tujia nationality and the Han nationality started during ancient times and became more frequent during the later Qing dynasty (1636–1911 AD). Xie Xuan Hua [13] explored the origin of Tujia ethnic nationality and their hybridization status with other ethnic groups by analyzing the distribution of haplotypes in the Y chromosome of the Tujia and found that their genetic structure was similar to those of Han. Zhou Jie’s study on 15 autosomal short tandem repeat loci polymorphisms revealed that the genetic makeup of the Tujia of Wufeng, Hubei province was similar to that of the Han of Hubei province (the Hubei Han group belongs to the central Han population) [14]; however if the genetic structure was compared between the Tujia and the central Han at HLA-A*31, HLA-A*30, DRB1*07 and DRB1*09 loci, a significant difference was found (*p*<0.05). This indicates that the genetic makeup of the Tujia and the central Han have both similarities and differences. The similarities come from gene hybridization and the differences relate to their ethnic origin. The genetic differences between the Hubei Tujia and the central Han are mainly owing to their isolated geographical location. Wufeng county is located in a mountainous area where transportation is very difficult and the resulting limited population mobility means that this area has a relatively pure genetic population. Investigating genetic diseases in the Wufeng area therefore has a significant meaning. Our result showed that gene alleles and haplotypes at HLA gene loci in the Hubei Wufeng population have a high incidence of polymorphisms. Studies of HLA haplotypes in the Hubei Tujia will help us to further understand their genetic background, evolution and origination. This will also help to enrich the genomic data resources of the Chinese population. This work is significant for further research on population genetics, genetic related diseases and vaccines. In addition HLA genetic analysis can help us to precisely evaluate the ratio of HLA matched individuals among donor pools for an organ recipient [15].

## Materials and Methods

### Ethical Statement

This project was approved by the Medical Ethics Committee of Wuhan University, China. All the individuals were healthy and provided informed consent and received a questionnaire. The investigation was conducted in accordance with humane and ethical research principles of Wuhan University, China. We confirm in our consent statement that consent was provided by 124 healthy individuals.

### Population Samples

The studied population consisted of 124 healthy, unrelated Tujia people chosen randomly from the Chengguan and Yuguan towns of Wufeng County, Hubei Province with the help of the local Maternity and Hygiene and Health Hospital. All of these individuals’ ancestors were born and lived in the Wufeng region of Hubei Province for at least three generations.

### DNA Extraction

Genomic DNA was isolated from whole blood containing ethylenediaminetetraacetic acid (EDTA), using a Genomic DNA Isolation Kit, according to the manufacturer’s instructions (BioVision Inc., Mountain View, CA) and frozen at −20°C until use. The concentration of DNA was 40–100 ng/µL, with the purity of the extracted DNA ranging from a 1.6 to a 1.85 OD value.

### DNA Typing of HLA Loci

HLA-A, HLA-B and HLA-DRB1 genotyping was performed on a Multi-Analyte Profiling system (xMAP) (Luminex HLA-SSO) using a WAKFlow HLA typing kit according to the manufacturer’s instructions. Please see ref. [16].

### Statistical Analysis

Allelic frequencies of HLA-A, -B and -DRB1 loci were estimated by the direct counting method. The haplotype frequencies are estimatives based on the alleles frequencies using the expectation maximization (EM) method with the Arlequin software package V3.11 (http://anthro.unige.ch/software/arlequin/) [17]. Tests of Hardy-Weinberg equilibrium were also carried out using this software. Linkage disequilibrium, the nonrandom association between two alleles at two different loci as defined by the delta (D’) coefficient, was calculated as described elsewhere [18]. Phylogenetic trees (dendrograms) were constructed based on allelic frequencies using the neighbor-joining (NJ) method with Nei distances using the phylogeny program Phylip (http://evolution.gs.washington.edu/phylip.html) [19]. Principal component analysis was processed using the SPSS 13.0 software package (SPSS Inc., Chicago, IL).
